# Clinical management of self-harming children and adolescents in the United Kingdom: a multicentre audit

**DOI:** 10.1192/j.eurpsy.2025.1542

**Published:** 2025-08-26

**Authors:** R. S. Goh, H. McAdam, F. Allman, G. Cheung, J. Alsop, S. Pandey, A. Hook, B. Perry, D. Codling, J. Randall, J. R. Harrison, K. Beck

**Affiliations:** 1 Public Health Policy Evaluation Unit, School of Public Health, Imperial College London, London; 2 University of Glasgow; 3 NHS Greater Glasgow and Clyde (Scotland Foundation School), Glasgow; 4 Newcastle University; 5 NCIC NHS FT, Newcastle; 6 Hull York Medical School; 7 York and Scarborough Teaching Hospitals NHS Foundation Trust, York; 8 University of Warwick, Warwickshire; 9 Luton and Dunstable NHS Foundation Trust, Luton; 10 Great Western Hospitals NHS Foundation Trust, Swindon; 11 University of Cambridge, Cambridge; 12 South London and Maudsley NHS Foundation Trust, London; 13 Translational and Clinical Research Institute, Faculty of Medical Science, Newcastle University, Campus for Ageing and Vitality, Newcastle; 14 King’s College London, London, United Kingdom

## Abstract

**Introduction:**

The risk of self-harm is highest in younger age groups, with increasing numbers of under-18s being admitted to hospital due to self-harm in recent years in the UK1,2. The National Institute for Health and Care Excellence (NICE) guidelines for self-harm in adolescents over eight was updated in September 2022 and reinforces the need for the proper initial management of adolescent self-harm3. To our knowledge, our study is the first UK national audit on the management of self-harm in adolescents presenting to the emergency department using the updated NICE guidelines.

**Objectives:**

To assess the clinical management of children and adolescents who present to the Emergency Department (ED) following self-harm, a cross-sectional, multicentre study was conducted within teaching hospitals affiliated with nine medical schools across England, Wales and Scotland.

**Methods:**

Data was retrospectively collected from ED records using consecutive sampling of individuals aged 8 to 17 years who presented with self-harm from 7 Sep-7 Nov 2022.

**Results:**

Records from 328 patients were included in the final analysis. Most patients were female (82.0%) and white (68.2%), with a mean age of presentation of 14.7 (σ = 1.58). The rate of positive responses to each question is available in Table 1. A ‘positive’ response is defined as a ‘yes’ response, rather than ‘no’ or ‘not documented’.

**Table 1.** Rate of compliance with audit criteria

Study	Intervention	Sample Size	Outcome Measure	Key Findings
Lamy et al. (2020)	SGAs (multiple RCTs)	>50 trials	ABC, CGI	Significant reduction in irritability (p<0.05)
Ghanizadeh et al. (2014)	Aripiprazole, Risperidone	59	ABC	Reduction in symptoms; fewer adverse events with aripiprazole
King et al. (2009)	Citalopram, Placebo	149	CGI, CY-BOCS	No significant benefit over placebo; more adverse effects

**Conclusions:**

This is the first study, to our knowledge, that investigates the management of self-harm in under 18s across the UK using the updated NICE guidelines. Some criteria may have been adhered to but not documented. The results from this study provide support for the further improvement of clinical practice in the management of self-harming children and adolescents.

**Disclosure of Interest:**

None Declared

